# P-68. Effect of hypoalbuminemia on blood culture clearance and outcomes in methicillin-susceptible Staphylococcus aureus bacteremia managed with antistaphylococcal penicillins or cefazolin

**DOI:** 10.1093/ofid/ofaf695.297

**Published:** 2026-01-11

**Authors:** Ryan W W Stevens, Daniel C DeSimone, Larry M Baddour, Kristin Cole, Kirstin Kooda, John Robinson, Dan Ilges

**Affiliations:** Mayo Clinic, Rochester, MN; Mayo Clinic, Rochester, MN; Mayo Clinic College of Medicine, Rochester, MN; Mayo Clinic, Rochester, MN; Mayo Clinic - Rochester, Rochester, Minnesota; Mayo Clinic Arizona, Phoenix, Arizona; Mayo Clinic Arizona, Phoenix, Arizona

## Abstract

**Background:**

Methicillin-susceptible *Staphylococcus aureus* bacteremia (MS-SAB) is treated with antistaphylococcal penicillins (ASP) or cefazolin. Altered pharmacokinetics in the setting of hypoalbuminemia may reduce the likelihood of therapeutic success in patients receiving highly protein bound beta-lactams. ASPs and cefazolin are 94% and 80% protein bound, respectively. We assessed the impact of hypoalbuminemia (≤2.5 g/dL) on clinical outcomes in patients with MS-SAB treated with ASPs or cefazolin.

**Table 1:**
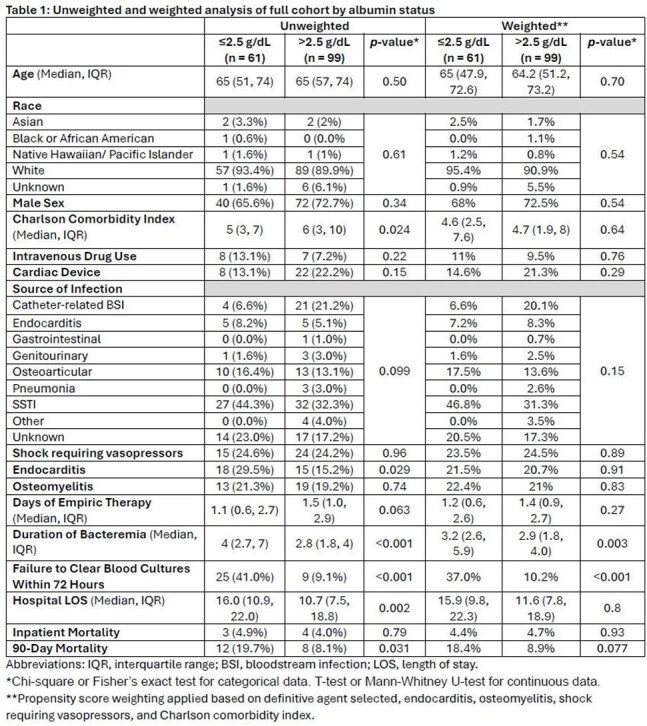
Unweighted and weighted analysis of full cohort by albumin status

**Table 2:**
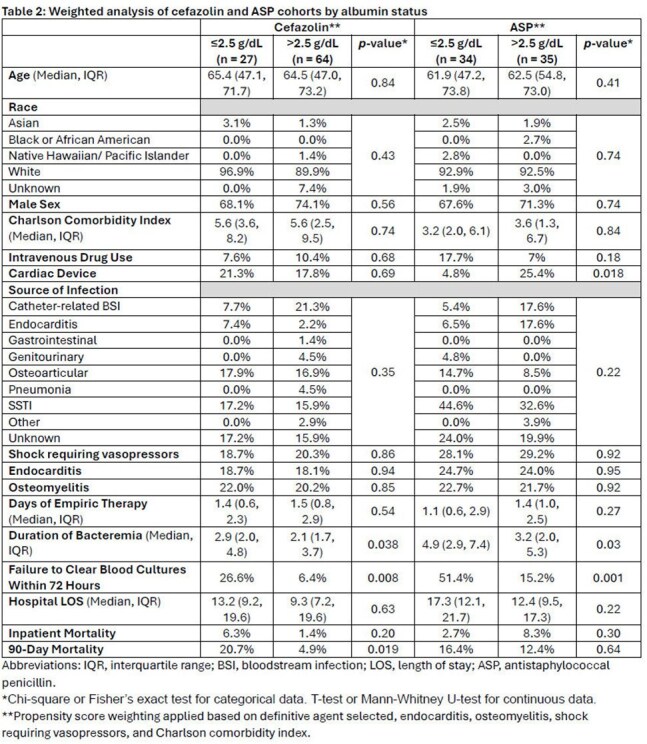
Weighted analysis of cefazolin and ASP cohorts by albumin status

**Methods:**

This single center, retrospective study included adults (≥18 years) admitted with community-acquired MS-SAB between 1/1/2006 and 12/31/2019 who received ASPs or cefazolin and had a serum albumin obtained between the date of first culture positivity and 72 hours of definitive therapy. Exclusion criteria included receipt of ≥5 days of MSSA active empiric therapy prior to definitive, polymicrobial bacteremia, antibiotics for an indication other than MS-SAB, or central nervous system infection. The primary outcome was failure to clear blood cultures within 72 hours of definitive therapy initiation. Propensity score weighting was used to adjust for baseline differences between cohorts.

**Results:**

160 patients were included with 61 (38.1%) being hypoalbuminemic (Table 1). In the propensity score weighted analysis, the primary outcome occurred in 37% of patients with an albumin ≤2.5 g/dL vs. 10.2% with an albumin >2.5 g/dL (p< 0.001). This finding was consistent in both ASP and cefazolin subgroups (Table 2). Hospital length of stay was longer in hypoalbuminemic patients but did not represent a statistically significant difference in the weighted overall analysis (15.9 vs. 11.6 days, p=0.8). There was no difference in inpatient mortality in any of the cohorts analyzed. 90-day all-cause mortality was higher in hypoalbuminemic patients only in the cefazolin subgroup analysis (20.7% vs 4.9%, p=0.019).

**Conclusion:**

The presence of hypoalbuminemia was associated with failure to clear blood cultures within 72 hours of definitive therapy initiation in patients with MS-SAB. While no significant difference in mortality was observed overall, the higher mortality in the cefazolin subgroup warrants further research on the impact of hypoalbuminemia on clinical outcomes in patients with MS-SAB.

**Disclosures:**

Larry M. Baddour, MD, UpToDate, Inc.: Royalty payments (authorship duties).

